# Supplementary material for: Assessing the predictive ability of computational epitope prediction methods on Fel d 1 and other allergens
Source: PLoS One. 2024 Aug 23;19(8):e0306254. doi: 10.1371/journal.pone.0306254 (PMC11343462; doi:10.1371/journal.pone.0306254)
Supplement: S1 Table — (DOCX) [file pone.0306254.s001.docx]

**S1 Table. Prediction results of a computational B cell epitope prediction tool for known allergen IgE epitopes**

|  | **MCC** | **Prediction of Epitopes** | | | **Prediction of Non-epitopes** | | |
| --- | --- | --- | --- | --- | --- | --- | --- |
|  |  | **PPV** | **Sensitivity** | **F1** | **NPV** | **Specificity** | **F1** |
| **ElliPro** | 0.035 | 0.29 | 0.52 | 0.38 | 0.74 | 0.52 | 0.61 |
| **BepiPred-2.0** | 0.058 | 0.32 | 0.52 | 0.39 | 0.74 | 0.55 | 0.63 |
| **BepiPred-3.0** | 0.129 | 0.36 | 0.46 | 0.41 | 0.76 | 0.67 | 0.71 |
| **Emini** | 0.049 | 0.32 | 0.39 | 0.35 | 0.73 | 0.66 | 0.7 |
| **Kolaskar** | 0.003 | 0.29 | 0.33 | 0.31 | 0.71 | 0.67 | 0.69 |
| **BepiPred-1.0** | -0.010 | 0.28 | 0.41 | 0.33 | 0.71 | 0.58 | 0.64 |
| **Parker** | -0.032 | 0.29 | 0.61 | 0.4 | 0.72 | 0.41 | 0.52 |
| **Karplus** | 0.014 | 0.28 | 0.52 | 0.36 | 0.69 | 0.45 | 0.54 |
| **Chou** | 0.015 | 0.29 | 0.67 | 0.41 | 0.72 | 0.34 | 0.46 |
| **Random** | 0.097 | 0.3 | 0.32 | 0.29 | 0.68 | 0.77 | 0.71 |
| **Number of  residues** |  | 3,553 (29 %) | | | 8,807 (71%) | | |
