# Supplementary material for: Assessing the predictive ability of computational epitope prediction methods on Fel d 1 and other allergens
Source: PLoS One. 2024 Aug 23;19(8):e0306254. doi: 10.1371/journal.pone.0306254 (PMC11343462; doi:10.1371/journal.pone.0306254)
Supplement: S2 Table — (DOCX) [file pone.0306254.s002.docx]

**S2 Table. T-cell epitope prediction results for food allergen IgE epitopes using ProPred and IEDB prediction methods.**

|  | **MCC** | | **Prediction of Epitopes** | | | | | | **Prediction of Non-epitopes** | | | | | |
| --- | --- | --- | --- | --- | --- | --- | --- | --- | --- | --- | --- | --- | --- | --- |
|  |  |  | **PPV** | | **Sensitivity** | | **F1** | | **NPV** | | **Specificity** | | **F1** | |
| **Allergen** | **ProPred** | **IEDB** | **ProPred** | **IEDB** | **ProPred** | **IEDB** | **ProPred** | **IEDB** | **ProPred** | **IEDB** | **ProPred** | **IEDB** | **ProPred** | **IEDB** |
| **Ana o 1** | 0.06 | 0.19 | 0.2 | 0.21 | 0.18 | 0.71 | 0.19 | 0.32 | 0.86 | 0.92 | 0.87 | 0.56 | 0.87 | 0.7 |
| **Ana o 2** | 0.12 | 0.22 | 0.35 | 0.31 | 0.2 | 0.71 | 0.25 | 0.43 | 0.8 | 0.87 | 0.9 | 0.56 | 0.85 | 0.68 |
| **Ara h 1** | 0.07 | 0.23 | 0.48 | 0.55 | 0.13 | 0.5 | 0.21 | 0.52 | 0.62 | 0.69 | 0.91 | 0.73 | 0.74 | 0.71 |
| **Ara h 2** | -0.03 | 0.21 | 0.28 | 0.53 | 0.1 | 0.31 | 0.14 | 0.39 | 0.68 | 0.72 | 0.88 | 0.87 | 0.77 | 0.79 |
| **Ara h 3** | 0.04 | 0.15 | 0.12 | 0.13 | 0.16 | 0.69 | 0.14 | 0.22 | 0.91 | 0.95 | 0.89 | 0.57 | 0.9 | 0.71 |
| **Arginine kinase** | 0.00 | 0.06 | 0.3 | 0.32 | 0.16 | 0.68 | 0.21 | 0.44 | 0.71 | 0.73 | 0.84 | 0.38 | 0.77 | 0.5 |
| **Beta-lactoglobulin** | 0.07 | 0.14 | 0.11 | 0.05 | 0.2 | 1 | 0.14 | 0.09 | 0.94 | 1 | 0.89 | 0.43 | 0.91 | 0.6 |
| **Mal d 1** | 0.03 | -0.05 | 0.18 | 0.13 | 0.1 | 0.43 | 0.13 | 0.2 | 0.86 | 0.83 | 0.92 | 0.5 | 0.89 | 0.63 |
| **Tropomyosin** | 0.05 | -0.13 | 0.15 | 0.08 | 0.18 | 0.43 | 0.16 | 0.14 | 0.89 | 0.83 | 0.87 | 0.38 | 0.88 | 0.53 |
| **Average** | 0.04 | 0.11 | 0.24 | 0.26 | 0.16 | 0.61 | 0.18 | 0.31 | 0.81 | 0.84 | 0.89 | 0.56 | 0.84 | 0.65 |
| **STD** | 0.04 | 0.13 | 0.12 | 0.19 | 0.04 | 0.21 | 0.04 | 0.15 | 0.11 | 0.11 | 0.02 | 0.16 | 0.07 | 0.09 |
